# High quality genome sequence and description of *Enterobacter mori* strain 5–4, isolated from a mixture of formation water and crude-oil

**DOI:** 10.1186/1944-3277-10-9

**Published:** 2015-02-27

**Authors:** Fan Zhang, Sanbao Su, Gaoming Yu, Beiwen Zheng, Fuchang Shu, Zhengliang Wang, Tingsheng Xiang, Hao Dong, Zhongzhi Zhang, DuJie Hou, Yuehui She

**Affiliations:** The Key Laboratory of Marine Reservoir Evolution and Hydrocarbon Accumulation Mechanism, School of Energy Resources, China University of Geosciences, Beijing, China; College of Chemistry and Environmental Engineering, Yangtze University, Jingzhou, China; College of Petroleum Engineering, Yangtze University, Jingzhou, China; State Key Laboratory for Diagnosis and Treatment of Infectious Diseases, Zhejiang University, Hangzhou, China; State Key Laboratory of Heavy Oil Processing, China University of Petroleum, Beijing, China

**Keywords:** *Enterobacter mori* strain 5–4, Formation water, Hydrocarbon degradation, Genome

## Abstract

*Enterobacter mori* strain 5–4 is a Gram-negative, motile, rod shaped, and facultatively anaerobic bacterium, which was isolated from a mixture of formation water (also known as oil-reservior water) and crude-oil in Karamay oilfield, China. To date, there is only one *E. mori* genome has been sequenced and very little knowledge about the mechanism of *E. mori* adapted to the petroleum reservoir. Here, we report the second *E. mori* genome sequence and annotation, together with the description of features for this organism. The 4,621,281 bp assembly genome exhibits a G + C content of 56.24% and contains 4,317 protein-coding and 65 RNA genes, including 5 rRNA genes.

**Electronic supplementary material:**

The online version of this article (doi:10.1186/1944-3277-10-9) contains supplementary material, which is available to authorized users.

## Introduction

The genus *Enterobacter* was created by Hormaeche and Edwards in 1960 [1]. Members of the genus were isolated mostly from the environment, in particular from plants and recognized as notorious plant pathogens, but were also frequently isolated from hospitals, notably in healthcare associated infections and recognized as opportunistic pathogens [2, 3]. Twenty-nine validly published species and 2 subspecies have previously been recorded in the genus *Enterobacter.* However, 17 of the validly named species have been subsequently reclassified as members of 11 other genera. As of Oct 2014, this genus contains only 10 species and two subspecies [4]. As of Oct, 2014, a total of 116 *Enterobacter* strains have been sequenced and 29 genome sequences were published [5–12], however, only one genome of *E. mori* isolated from diseased mulberry roots has been sequenced [13]. *E. mori* strain 5–4 is a Gram-negative, motile, rod shaped, and facultatively anaerobic bacterium, isolated from a crude-oil well. It is worthy of note that *E. mori* strain 5–4 is capable of degrading petroleum (Additional file 1). In order to elucidate comprehensive alkane degradation pathways and adaption mechanism in *E. mori* strain 5–4, whole-genome sequence analysis was thus conducted. Here, we present a summary classification and a set of features for *E. mori* strain 5–4, together with the description of the genomic sequencing and annotation.

### Classification and features

**Figure 1:**
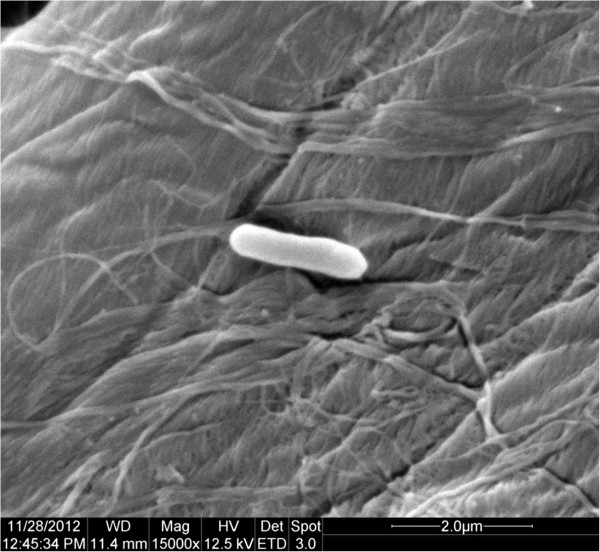
Scanning electron micrograph of cells of *Enterobacter mori* strain 5–4 bar: 2.0 μm.

A formation water sample was collected from Karamay Oilfield, Xinjiang, China, in 2012. The water sample was preserved at -80°C immediately after collection and sent to the lab. *E. mori* strain 5–4 was isolated after cultivation on LB agar medium at 37°C. The optimum temperature for growth is 35°C, with a temperature range of 4-45°C (Table 1). Growth occurs under aerobic condition. Grows at pH 5.5-10.0, and optimally at pH 7.0. Cell morphology was examined by using scanning electron microscopy (Quanta 200, FEI Co., USA). Colonies are light yellow, smooth, circular with entire margins, with a diameter ranging 0.3-0.8 μm, and from 0.6 to 1.8 μm long (Figure 1). Themethyl red test is negative. H_2_S and indole are not produced. Casein and starch are not hydrolysed; gelatin is hydrolysed. Sorbitol, glycerol, tetradecane and hexadecane are utilized as the carbon source, while lactose, rhamnose, glucose, maltose, cellobiose, galactose, raffinose and sucrose are not utilized. Nitrite sodium and ammonium chloride are utilized, while nitrate sodium is not reduced. Antimicrobial susceptibility test showed that this strain is susceptible to ampicillin, tetracycline, erythromycin and gentamicin, and resistant to kanamycin.

**Table 1:** **Classification and general features of** ***Enterobacter mori*** **strain 5–4 according to the MIGS recommendations** [14]

MIGS ID	Property	Term	Evidence code ^a^
	Classification	Domain *Bacteria*	TAS [15]
		Phylum *Proteobacteria*	TAS [16]
		Class *Gammaproteobacteria*	TAS [17, 18]
		Order *Enterobacteriales*	TAS [19]
		Family *Enterobacteriaceae*	TAS [20–22]
		Genus *Enterobacter*	TAS [20, 23, 24]
		Species *Enterobacter mori*	
		Strain: Strain 5-4	IDA
	Gram stain	Negative	IDA
	Cell shape	Rod	IDA
	Motility	Motile	IDA
	Sporulation	Non-sporulating	IDA
	Temperature range	4-45°C	IDA
	Optimum temperature	35°C	IDA
	pH range; Optimum	Unknown	IDA
	Carbon source	Sorbitol, glycerol, tetradecane and hexadecane	IDA
MIGS-6	Habitat	Environment	IDA
MIGS-6.3	Salinity	Growth in 0% ~ 7% NaCl	IDA
MIGS-22	Oxygen requirement	Aerobic	IDA
MIGS-15	Biotic relationship	Free living	IDA
MIGS-14	Pathogenicity	Unknown	IDA
MIGS-4	Geographic location	Karamay, China	IDA
MIGS-5	Sample collection	2012	IDA
MIGS-4.1	Latitude	45°62’N	IDA
MIGS-4.2	Longitude	85°02’E	
MIGS-4.4	Altitude	460 m	IDA

^a^Evidence codes - IDA: Inferred from Direct Assay; TAS: Traceable Author Statement (i.e., a direct report exists in the literature); NAS: Non-traceable Author Statement (i.e., not directly observed for the living, isolated sample, but based on a generally accepted property for the species, or anecdotal evidence). These evidence codes are from the Gene Ontology project [25].

**Figure 2:**
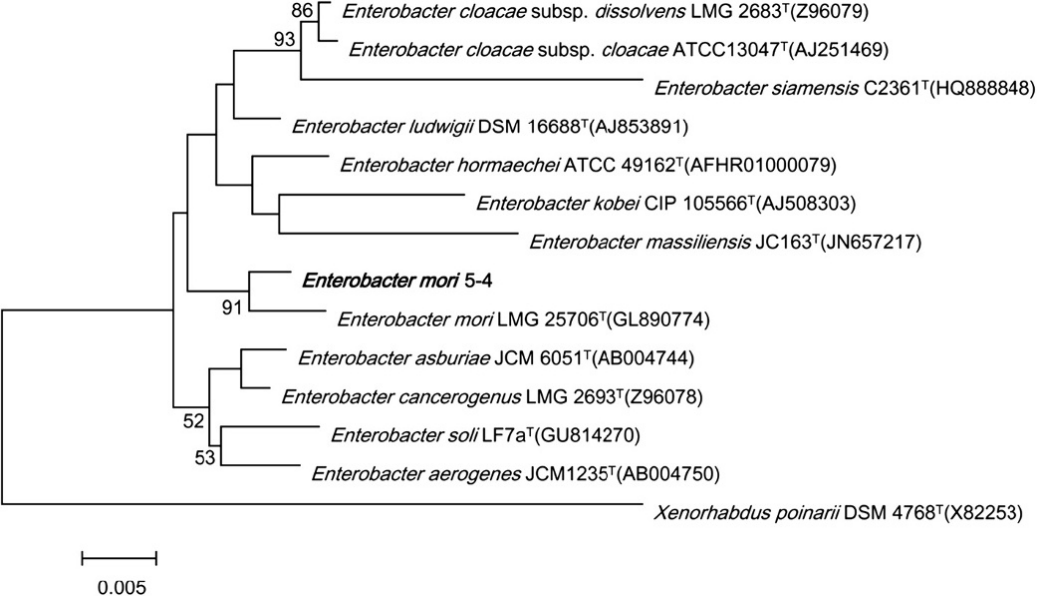
Phylogenetic tree highlighting the position of *E. mori* 5*–*4 relative to other type strains within the genus *Enterobacter.* The strains and their corresponding GenBank accession numbers for 16S rRNA genes are shown following the organism names. Bootstrap consensus trees were inferred from 100 replicates, only bootstrap values > 50% were indicated. *Xenorhabdus poinarii* DSM 4768^T^ was used as anoutgroup. The scale bar, 0.0005 substitutions per nucleotide position.

A comparative taxonomic analysis was conducted based on the 16S rRNA nucleotide sequence. The representative 16S rRNA nucleotide sequence of *Enterobacter mori* strain 5–4 was compared against the most recent release of the EzTaxon-e database [26]. CLUSTAL W was used to generate alignments with comparative sequences collected from EzTaxon-e database [27]. The alignments were trimmed and converted to the MEGA 6.06 format before phylogenetic analysis. Phylogenetic inferences were made using Neighbor-joining method based on Tamura-Nei model within the MEGA 6.06 [28]. Phylogenetic tree indicated the taxonomic status of strain 5–2, clearly classified into the same branch with species *E. mori* type strain LMG 25706^T^ (Figure 2).

### Genome sequencing information

#### Genome project history

*E. mori* strain 5–4 was selected for whole genome sequencing on the consideration of its potential relevance to microbial enhanced oil recovery (MEOR). The genome project is deposited in the Genome On Line Database and the draft genome sequence is deposited in GenBank under the accession JFHW00000000 and consists of 36 contigs. A summary of the project information and its association with MIGS version 2.0 compliance are shown in Table 2[14].

**Table 2:** **Project information**

MIGS ID	Property	Term
MIGS-31	Finishing quality	High-quality draft
MIGS-28	Libraries used	One pair-end 450 bp library
MIGS-29	Sequencing platforms	Illumina HiSeq 2000
MIGS-31.2	Fold coverage	358.0 × (based on 450 bp library)
MIGS-30	Assemblers	Velvet 1.2.07
MIGS-32	Gene calling method	Glimmer 3.0
	Locus Tag	AA74
	Genbank ID	JFHW00000000
	Genbank Date of Release	April 2, 2014
	GOLD ID	Gi0064796
	BIOPROJECT	PRJNA224116
	Project relevance	Industrial
MIGS-13	Source Material Identifier	CGMCC9982

### Growth conditions and DNA isolation

*E. mori* strain 5–4 was x-Bertani Broth. Cells in late-log-phase growth were harvested and lysed by EDTA, lysozyme, and detergent treatment, followed by proteinase K and RNase digestion. Genomic DNA was extracted using the DNeasy blood and tissue kit (Qiagen, Germany), according to the manufacturer’s recommended protocol. The quantity of DNA was measured by the NanoDrop Spectrophotometer and Cubit. Then 10 μg of DNA was sent to BGI (Shenzhen, China) for sequencing on a Hiseq2000 (Illumina, CA) sequencer.

#### Genome sequencing and assembly

Genomic DNA sequencing of *E. mori* strain 5–4 was performed using Solexa paired-end sequencing technology (HiSeq2000 system, Illumina). One DNA library was generated (450 bp insert size, with Illumina adapter at both end, detected by Agilent DNA analyzer 2100), then sequencing was performed with a 2 x 100 bp pair end sequencing strategy. Finally, a total of 6,652.30 M bp data was produced and quality control was performed with the following criteria: 1) Reads linkaged to adapters at both end were considered as sequencing artifacts then removed. 2) Bases with quality index lower than Q20 at both end was trimmed. 3) Reads with ambiguous bases (N) were removed. 4) Single qualified reads were discarded (In this situation, one read is qualified but its mate is not). Filtered 687.39 M clean reads were assembled into scaffolds using the Velvet version 1.2.07 with parameters “-scaffolds no” [29], then we use a PAGIT flow [30] to prolong the initial contigs and correct sequencing errors to arrive at a set of improved scaffolds.

#### Genome annotation

Predict genes were identified using Glimmer version 3.0 [31], tRNAscan-SE version 1.21 [32] was used to find tRNA genes, whereas ribosomal RNAs were found by using RNAmmer version 1.2 [33]. To annotate predict genes, we used HMMER version 3.0 [34] to align genes against Pfam version 27.0 [35] (only pfam-A was used) to find genes with conserved domains. KAAS server [36] was used to assign translated amino acids into KEGG Orthology [37] with SBH (single-directional best hit) method. Translated genes were aligned with COG database [38, 39] using NCBI blastp (hits should have scores no less than 60, e value is no more than 1e-6). To find genes with hypothetical or putative function, we aligned genes against NCBI nucleotide sequence database database (nt database was downloaded at Sep 20, 2013 ) by using NCBI blastn, only if hits have identity no less than 0.95, coverage no less than 0.9 , and reference gene had annotation of putative or hypothetical. To define genes with singnal peptide, we use SignaIP version 4.1 [40] to identify genes with signal peptide with default parameters. TMHMM 2.0 [41] was used to identify genes with transmembrane helices.

#### Genome properties

The draft genome sequence of *E. mori* strain 5–4 was assembled into 36 scaffolds with a assembly genome size of 4,621,281 bp and a G + C content of 56.2% (*N*_*50*_ is 358,174 bp). These scaffolds contain 4317 coding sequences (CDSs), 60 tRNAs (excluding 0 Pseudo tRNAs) and incomplete rRNA operons (3 small subunit rRNA and 2 large subunit rRNAs). A total of 980 protein-coding genes were assigned as putative function or hypothetical proteins. 3625 genes were categorized into COGs functional groups (including putative or hypothetical genes). The properties and the statistics of the genome are summarized in Table 3 and Table 4.

**Table 3:** **Genome statistics**

Attribute	Value	% of total^a^
Genome size (bp)	4,621,281	100.00
DNA Coding region (bp)	4,117,467	89.10
DNA G + C content (bp)	2,599,117	56.24
DNA scaffolds	36	
Total genes	4,322	100.00
Protein-coding genes	4,317	99.88
RNA genes	65	1.51
Pseudo genes	17	0.39
Genes with function prediction	980	22.67
Genes assigned to COGs	3,625	83.87
Genes assigned to Pfam domains	3,995	92.43
Genes with signal peptides	420	9.72
Genes with transmembrane helices	1,085	25.10
CRISPR repeats	1	0.023

^a^The total is based on either the size of the genome in base pairs or the total number of protein coding genes in the annotated genome.

**Table 4:** **Number of genes associated with the general COG functional categories**

Code	Value	% age	Description
J	202	4.68	Translation, ribosomal structure and biogenesis
A	1	0.02	RNA processing and modification
K	400	9.27	Transcription
L	149	3.45	Replication, recombination and repair
B	1	0.02	Chromatin structure and dynamics
D	59	1.37	Cell cycle control, mitosis and meiosis
V	146	3.38	Defense mechanisms
T	228	5.28	Signal transduction mechanisms
M	266	6.16	Cell wall/membrane biogenesis
N	136	3.15	Cell motility
U	130	3.01	Intracellular trafficking and secretion
O	176	4.08	Posttranslational modification, protein turnover, chaperones
C	295	6.83	Energy production and conversion
G	499	11.56	Carbohydrate transport and metabolism
E	604	13.99	Amino acid transport and metabolism
F	94	2.18	Nucleotide transport and metabolism
H	230	5.33	Coenzyme transport and metabolism
I	120	2.78	Lipid transport and metabolism
P	421	9.75	Inorganic ion transport and metabolism
Q	134	3.10	Secondary metabolites biosynthesis, transport and catabolism
R	720	16.68	General function prediction only
S	361	8.36	Function unknown
-	333	7.71	Not in COGs

The total is based on the total number of protein coding genes in the annotated genome.

### Genome comparison

**Figure 3:**
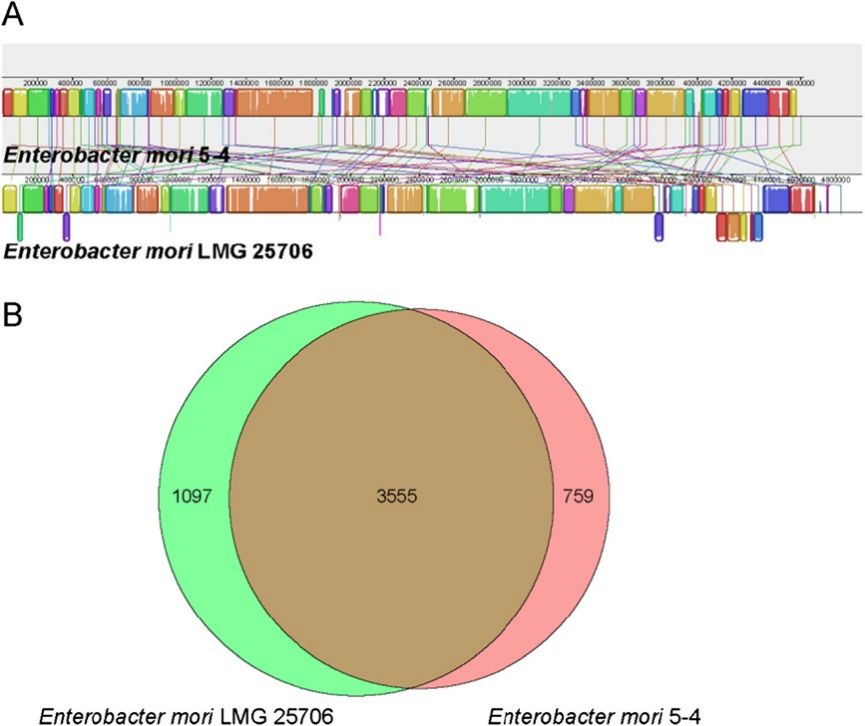
**Genome comparison between** ***E. mori*** **5–4** **and** ***E. mori*** **LMG 25706** ^**T**^ **. (A)**. Alignment is represented as local colinear blocks (colored) filled with a similarity plot. Height of the similarity plot indicates nucleotide identity of both assemblies; **(B)**. Numbers inside the Venn diagrams indicate the number of genes found to be shared among the indicated genomes.

Genome alignment between *E. mori* 5–4 (JFHW00000000) and *E. mori* type strain LMG 25706 T (AEXB00000000) was performed by using Mauve [42]. Orthology identification was carried out by a modified method introduced by Lerat [43]. Genome alignment showed that some functional regions are highly homologous between these two assemblies. The alignment also reveals some discrepancies between them, some short stretches of LMG 25706 T genome absent from the contigs in 5–4 (Figure 3A). However, two alkane 1-monooxygenase, one alkanesulfonate monooxygenase, one putative alkanesulfonate transporter, one putative sulfate permease and one alkanesulfonate transporter permease subunit were identified in the genome. Alkane 1-monooxygenase was found as one of the key enzymes responsible for the aerobic transformation of n-alkanes [44]. Moreover, alkanesulfonate monooxygenase and alkanesulfonate transporter may be responsible for organosulfur compound degradation [45]. Comparison of these two strains revealed the presence of a large core-genome (Figure 3B). They shared 3555 CDS in the genome. In addition, 759 CDS from the 5–4 genome were classified as unique, while 1097 CDS from the LMG 25706 T genome were classified as unique. Our genomic data will provide an excellent platform for further improvement of this organism for potential application in bioremediation.

## Conclusions

Here, we report the second draft genome sequence and description of *E. mori*, which was isolated from a mixture of formation water and crude-oil. The genome revealed two alkane 1-monooxygenase, one alkanesulfonate monooxygenase, one putative alkanesulfonate transporter, one putative sulfate permease and one alkanesulfonate transporter permease subunit. Our genomic data of strain 5-4 provide a vast pool of genes involved in hydrocarbon degradation and an excellent platform for further improvement of this organism for potential application in bioremediation of oil-contaminated environments. And further comparative genomic study between stain 5-4 and other *Enterobacter* strains will give us a better understanding of the evolution of environmental bacteria towards industrial application.

## Electronic supplementary material

Additional file 1: Figure S1: Crude-oil and liquid paraffin degradation of *E. mori* 5*–*4. (A) Bio-degradation of crude-oil by *E. mori* 5*–*4 after 4-days incubation; (B) Negative control of crude-oil degradation; (C) Bio-degradation of liquid paraffin by *E. mori* 5*–*4 after 4-days incubation; (D) Negative control of liquid paraffin degradation. (DOCX 2 MB)
