# Supplementary material for: High quality genome sequence and description of Enterobacter mori strain 5–4, isolated from a mixture of formation water and crude-oil
Source: Stand Genomic Sci. 2015 Feb 27;10:9. doi: 10.1186/1944-3277-10-9 (PMC4940761; doi:10.1186/1944-3277-10-9)
Supplement: Supplementary file 1 — Additional file 1: Figure S1: Crude-oil and liquid paraffin degradation of E. mori 5–4. (A) Bio-degradation of crude-oil by E. mori 5–4 after 4-days incubation; (B) Negative control of crude-oil degradation; (C) Bio-degradation of liquid paraffin by E. mori 5–4 after 4-days incubation; (D) Negative control of liquid paraffin degradation. (DOCX 2 MB) [file 40793_2014_38_MOESM1_ESM.docx]

**Figure S1.** Crude-oil and liquid paraffin degradation of *E. mori* 5-4. (A) Bio-degradation of crude-oil by *E. mori* 5-4 after 4-days incubation; (B) Negative control of crude-oil degradation; (C) Bio-degradation of liquid paraffin by *E. mori* 5-4 after 4-days incubation; (D) Negative control of liquid paraffin degradation.

**
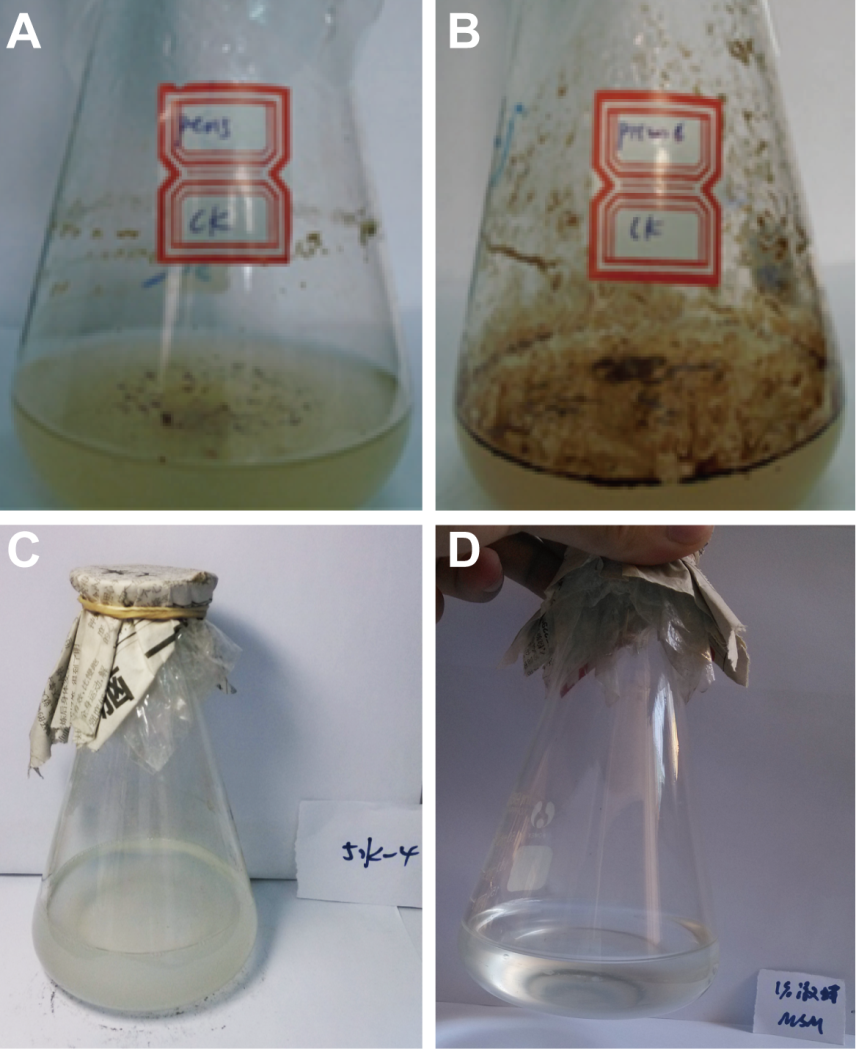
**
